# Letter from Dr. Isaac I. Greenwood to the Baltimore Editor

**Published:** 1841-12

**Authors:** Isaac I. Greenwood


### ARTICLE II.

### *Letter from* DR. ISAAC I. GREENWOOD, *Surgeon Dentist, of New York, to the Baltimore Editor.*

DEAR SIR:—Permit me to state a case for your consideration of disease of the dexter antrum, or great maxillary sinus, which presented itself to me some two years since, with its cure as performed under my treatment at the time.

During the years 1838-9, a young man about twenty two years of age, applied to me to have a medium molar tooth of the upper maxillary filled. I cleaned out the cavity, (the pulp and branches of the medullary canal having to appearance been destroyed for some time,) and performed the operation, as I thought, perfectly satisfactory both to the patient and myself, he complaining of no inconvenience at the time, and seeming well pleased with the operation. In about a week after, he returned, complaining of a heavy pain in the head and temples, telling me at *this time* that the tooth had bothered him (as he expressed it) some time before I had plugged it very much. I took out the filling and found a very fetid smell to come from the aperture, and a small discharge of discoloured watery humour. I placed some raw cotton saturated with kreosote and laudanum into the cavity, and sent him away. He however returned the next day, and complained of a sensation as if molten lead was in his cheek-bone, and that he could not use his right arm (he was by trade a carpenter) on account of its weakness, and the constant pains and heaviness in his head, and the muscles of the cheek on that side, when he attempted to drive a plane in smoothing of boards; he likewise felt a sleepy sensation, but could get no rest from the continued benumbing feeling accompanied by twitchings in the region of the eye and the muscles of the face. From what he told me I knew it was useless to allow the diseased organ to remain and accordingly I extracted it. Considerable pus followed the operation, accompanied by a somewhat sanguineous discharge from the alveolus. This being arrested, I proposed to him to allow me to open an aperture into the antrum highmorianum, which he consented to, and taking a straight three side headed perforator, and driving it gently, and by a spiral rotary motion, following one of the facial alveolar fossæ about an inch or more into the great sinus. The result was, immediate relief and a considerable flux of fetid dark coagulated blood and matter, or pus. The orifice made by the perforator was about the size of a common pipe stem, such as used in smoking: this I made somewhat larger, and when the oozing had in a measure abated, I wrapped some raw cotton round the end of a piece of straight iron wire, and dipping it into a mixture of muriatic acid, kreosote and aqua rosæ, I forced it into the antrum, and giving it a rotary motion from side to side, endeavoured to mix up the pus, &c. with the solution introduced. This I did two or three times, and each time after the introduction of the mixture as applied, I used dry raw cotton alone, to endeavour to absorb as much of the impurities as possible. I then placed some raw cotton, saturated as above into the cavity, to remain there, and sent him home, telling him to return the next morning. When he came, I again cleaned out the antrum with dry raw cotton and the same mixture as before, although not so strong as that used the time previous, and required him to come in *three* days, as he now felt much better. He did so, when I used a mixture of the acetate of plumbi and aqua rosæ, and treated the cavity the same as before, telling him to keep the body open by gentle aperients. He had been enabled to sleep and felt much better. There was a continual discharge from the sinus. When he called in a week after, he was much better, in good spirits, and enabled to use his arm somewhat, but not satisfactorily; and could attend to his trade, but not much. I advised him to continue the aperients and syringe the cavity frequently with *suds* made from tepid soft water and old Castile soap, and to bathe the external surface of the temple and cheek with tepid water and vinegar and laudanum in equal portions; which he promised to do, and as I heard no more of him conclude the cure was effected.

In the instance of a case treated by my father, John Greenwood, nothing was prescribed but *tepid Castile soap suds*, to be repeatedly injected into the antrum by means of a syringe, until cured, which was effected in a short time after having applied the remedy; and the gentleman is now living to attest the perfect propriety of the prescription, in the complete restoration of the healthiness of the parts diseased.

With sentiments of sincere respect and esteem, dear sir, yours respectfully, ISAAC I. GREENWOOD.

